# Physical realizations of inerter and inerter-based vibration control

**DOI:** 10.1016/j.heliyon.2024.e35870

**Published:** 2024-08-06

**Authors:** Yuehao Li, Niaoqing Hu, Yi Yang, Zhe Cheng, Zhengyang Yin, Zuanbo Zhou, Jiangtao Hu

**Affiliations:** a College of Intelligence Science and Technology, National University of Defense Technology, Changsha, 410073, China; b Laboratory of Science and Technology on Integrated Logistics Support, NUDT, Changsha, 410073, China

**Keywords:** Inerter, Physical realizations, Vibration control, Review

## Abstract

Vibration control is extremely important for countless mechanical systems. Inerter is a two-terminal dynamic element proposed in 2002, based on analogy between mechanical system and electric system. Dynamic characteristic of an ideal inerter is pure inertia. Force applied on each terminal of an inerter is directly proportional to relative acceleration of two terminals. Since inerter was put forward, it has made significant progress in vibration control systems. The paper is a review about physical realizations of inerter as well as inerter-based vibration control. Physical realizations and applications in vibration control of inerter are focused. First, the develop of inerter and typical physical realizations of inerter are introduced. The normative derivation processes based on Lagrange equation method of the dynamic relationships in the different inerters are summarized. And then, three categories of common inerter-based vibration control systems are explained. Finally, research trend of physical realizations of inerter are summarized, and the possible researches on inerter-based vibration control are discussed.

## 1 Introduction

Vibration exists in various mechanical systems. To avoid the effect of harmful vibration and collect the energy of vibration, vibration control is widely used and extremely important for countless mechanical systems. Inerter is a two-terminal dynamic element, proposed by Smith [1] in 2002. It was proposed based on analogy between mechanical system and electric system [2]. Dynamic characteristic of an ideal inerter is pure inertia. Force applied on each terminal of an inerter is directly proportional to relative acceleration of two terminals. Dynamic characteristic of an ideal inerter is similar to an ideal capacitor. Therefore, based on analogy between mechanical system and electric system, an ideal inerter can correspond to an ideal capacitor. Before the inerter is proposed, there were no mechanical elements that could exactly correspond to a two-terminal capacitor. And the mechanical element, mass, only corresponds to “grounded capacitor”. On account of develop of inerter, mechanical network is almost complete.

In past 20 years, inerter has made significant progress in vibration control systems. Categories of inerters were applied in different systems, such as the vehicle suspension system [[3], [4], [5], [6], [7], [8], [9], [10], [11], [12], [13], [14], [15], [16], [17], [18], [19], [20], [21], [22], [23], [24], [25], [26], [27], [28], [29], [30], [31], [32], [33], [34], [35], [36], [37], [38], [39], [40]], the structure vibration control system of buildings [[41], [42], [43], [44], [45], [46], [47], [48], [49], [50], [51], [52], [53], [54], [55], [56], [57], [58], [59], [60], [61], [62], [63], [64], [65], [66], [67], [68], [69], [70], [71], [72], [73], [74], [75]], vibration control system of bridges [[76], [77], [78], [79], [80], [81], [82], [83], [84], [85], [86], [87], [88], [89], [90], [91], [92], [93], [94], [95], [96], [97], [98], [99], [100], [101], [102], [103]], the landing gear buffer system [[104], [105], [106]], vibration energy harvesting device [21,52,91,95,[107], [108], [109], [110], [111], [112], [113], [114]] and energy sink [[115], [116], [117], [118], [119], [120], [121], [122], [123], [124], [125], [126], [127], [128], [129], [130]], etc. Inerter has provided different routes to the vibration control problems of these systems, compared with the traditional solutions. And inerters have been proved to be effective in many applications. Moreover, researches of the inerter increase by years. Various physical inerters have appeared, such as rack-and-pinion inerter [1,3,6,16,42,46,77,94,[131], [132], [133], [134], [135], [136], [137], [138], [139], [140]], ball-screw inerter [4,[7], [8], [9],^27^,^38^,^39^,^41^,^57^,^62^,^80^,^82^,^109^,[141], [142], [143], [144], [145], [146], [147], [148], [149], [150], [151], [152], [153], [154], [155], [156], [157], [158], [159]], hydraulic inerter [17,160,161], fluid inerter [20,23,48,54,[162], [163], [164], [165], [166], [167], [168], [169]], living-hinge inerter [[170], [171], [172]], electromechanical inerter [11,26,31,34,38,39,43,69,80,95,107,109,113,156,[173], [174], [175], [176], [177], [178], [179], [180], [181], [182], [183], [184], [185]], and novel nonlinear inerter [[186], [187], [188], [189]], etc. Physical realizations of inerter, i.e. the actual inerters, are different physical devices used for specific mechanical systems. The dynamic characteristics and dynamic models of different physical realizations of inerter vary greatly.

This paper is a review about the physical realizations of inerter as well as inerter-based vibration control. Physical realizations and applications in vibration control of inerter are focused. The main contribution of this review is to synthesize the various physical realization forms of inerter and the latest progress, indicating three main vibration control methods based on inerter, which are not restricted to the field of civil engineering or mechanical engineering. The normative derivation processes based on Lagrange equation method of the dynamic relationships in the different inerters are summarized. This review article will be quite beneficial to the scholars who are interested in inerter. First, the develop of inerter and typical physical realizations of inerters are introduced in Section 2. The normative derivation processes based on Lagrange equation method of the dynamic relationships in the different inerters are summarized. And then, three categories of common inerter-based vibration control systems are explained in Section 3. Finally, in conclusions part, the trend of the physical realizations of inerter are summarized, and the possible researches on inerter-based vibration control are discussed.

## 2 Develop and physical realizations of inerter

### 2.1 Develop of inerter

For a long time, scholars have devoted themselves to analyzing the similarities between mechanical system and electrical system. And force-current analogy theory between mechanical system and electric system has been founded. In analogy theory, force corresponds to current, and velocity corresponds to voltage, a spring corresponds to an inductor, a damper corresponds to a resistor, and mass corresponds to “grounded capacitor”. Before the inerter is proposed, there were no mechanical elements that could exactly correspond to a two-terminal capacitor. And this fact was changed in 2002. In the year of 2002, a novel kind of two-terminal dynamic element was proposed by Smith [1].

Actually, inerter is a concept proposed by Smith. Before this, rotational mass damper or mass pump have been developed and adopted on vibration control of pipes, which has the similar idea, but not yet named after “inerter” before Smith. And the detailed historical perspective of inerter-like devices can be found in Ref. [190]. Smith proposed strict definition and principles of inerter.

The definition of inerter is that “ideal inerter is a mechanical two-node, one-port device with the property that the equal and opposite force applied at the nodes is proportional to the relative acceleration between the nodes” [1]. Dynamic characteristic of an ideal inerter is pure inertia. And dynamic characteristic of an ideal inerter is similar to an ideal capacitor. Based on force-current analogy theory between mechanical system and electric system, an ideal inerter can correspond to an ideal capacitor. Because of the inerter, the mechanical network is almost complete.

**Fig. 1:**
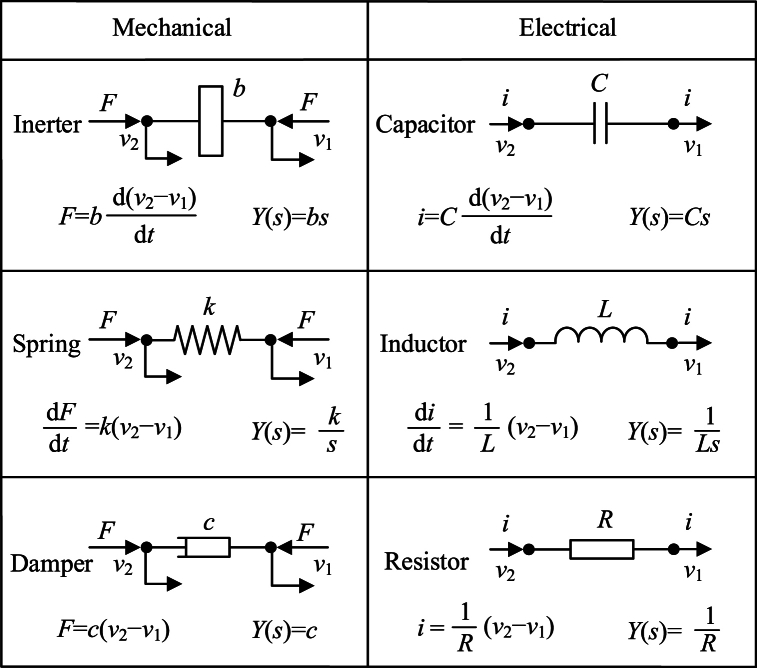
The analogy theory between mechanical system and electric system [1,9].

Completed force-current analogy theory between mechanical system and electric system is shown as Fig. 1. *F* is force on each terminal of a mechanical element, and *i* is current flowing through each terminal of an electrical element. *v*_1_ and *v*_2_ are the voltages of two terminals of an electrical element or the velocities of two terminals of a mechanical element. *b* is inertance of an inerter, and *C* is capacitance of a capacitor. *k* is stiffness of a spring, and *L* is inductance of an inductor. *c* is damping coefficient of a damper, and *R* is resistance of a resistor. *Y*(s) is admittance of a mechanical or electric element, defined by Smith in Ref. [1]. And *t* is the time. Based on force-current analogy theory between mechanical system and electrical system, different kinds of novel mechanical networks and devices are proposed by researchers.

### 2.2 Physical realizations of inerter

After the inerter's develop, various categories of inerter devices have been put forward and realized, such as rack-and-pinion inerter [1,3,6,16,42,46,77,94,[131], [132], [133], [134], [135], [136], [137], [138], [139], [140]], ball-screw inerter [4,[7], [8], [9],^27^,^38^,^39^,^41^,^57^,^62^,^80^,^82^,^109^,[141], [142], [143], [144], [145], [146], [147], [148], [149], [150], [151], [152], [153], [154], [155], [156], [157], [158], [159]], hydraulic inerter [17,160,161], fluid inerter [20,23,48,54,[162], [163], [164], [165], [166], [167], [168], [169]], living-hinge inerter [[170], [171], [172]], electromechanical inerter [11,26,31,34,38,39,43,69,80,95,107,109,113,156,[173], [174], [175], [176], [177], [178], [179], [180], [181], [182], [183], [184], [185]], and novel nonlinear inerter [[186], [187], [188], [189]], etc. Physical realizations of inerter, i.e. the actual inerters, are different physical devices used for specific mechanical systems. The dynamic characteristics and dynamic models of different physical realizations of inerter vary greatly. It is necessary to introduce and analyze different categories of physical realizations of inerter. The normative derivation processes based on Lagrange equation method of the dynamic relationships in the different inerters are summarized. To express energy intuitively, we use velocity *v* as a basic function, in the normative derivation processes based on Lagrange equation method.

#### 2.2.1 Ball-screw inerter

The ball-screw inerter [4,[7], [8], [9],^27^,^38^,^39^,^41^,^57^,^62^,^80^,^82^,^109^,[141], [142], [143], [144], [145], [146], [147], [148], [149], [150], [151], [152], [153], [154], [155], [156], [157], [158], [159]] is another kind of typical mechanical inerter and the early physical realization of inerter. Ball-screw inerter is nearly the most widely researched and used inerter. There are two kinds of basic realization structures of ball-screw inerters: nut rotary type ball-screw inerter and screw rotary type ball-screw inerter.

##### 2.2.1.1 The screw rotary type ball-screw inerter

**Fig. 2:**
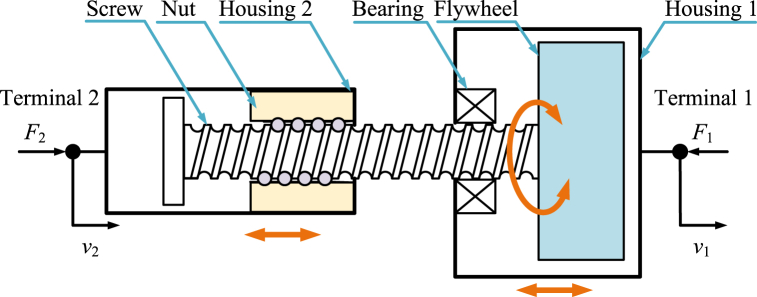
Diagram of the typical screw rotary type ball-screw inerter [7].

Diagram of a typical screw rotary type ball-screw inerter [7] is shown as Fig. 2. The typical screw rotary ball-screw inerter mainly consists of a pair of ball nut and screw, a flywheel, bearings, and two housings. Housing 2 is terminal 2, and housing 1 is terminal 1. The housing 2 and the housing 1 can move horizontally relatively. The nut is fixed with the housing 2. The flywheel is fixed with the screw. The nut can drive the screw and the flywheel to rotate at the same angular velocity. Moment of inertia of flywheel is far more than moment of inertia of screw and parts of bearings fixed to the screw. As a result, kinetic energy of the typical screw rotary type ball-screw inerter is mainly supplied by the angular kinetic energy of flywheel.

##### 2.2.1.2 The nut rotary type ball-screw inerter

**Fig. 3:**
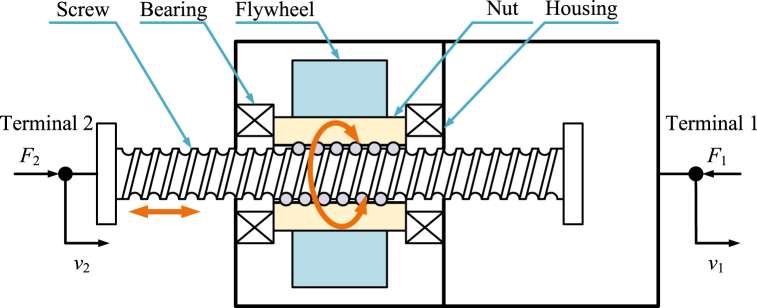
Diagram of a typical nut rotary type ball-screw inerter [4,154,155].

Diagram of a typical nut rotary type ball-screw inerter [4] is shown as Fig. 3. A typical nut rotary ball-screw inerter mainly consists of a pair of ball nut and screw, a flywheel, bearings, as well as a housing. Screw is terminal 2, and housing is terminal 1. Housing and screw can move horizontally relatively. Flywheel is fixed to ball nut. Screw can drive the nut and the flywheel to rotate at the same angular velocity. Moment of inertia of flywheel is far more than moment of inertia of nut and parts of bearings fixed to nut. As a result, kinetic energy of a typical nut rotary type ball-screw inerter is mainly supplied by the angular kinetic energy of flywheel.

Only considering the angular kinetic energy of the flywheel, ignoring the friction and other factors, the dynamic relationship in a typical nut rotary type ball-screw inerter in Fig. 3 and a typical screw rotary type ball-screw inerter in Fig. 2, is expressed as Eq. (1).

(1) $$F_{2}=F_{1}=b_{I}\left({\overset{．}{v}}_{2}-{\overset{．}{v}}_{1}\right),b_{I}=I_{F}{\left(\frac{2\pi }{P_{b}}\right)}^{2}$$

where *P*_b_ is lead of ball-screw pair.

The normative derivation process based on Lagrange equation method of dynamic relationships in a typical ball-screw inerter is shown below. Kinetic energy of a typical ball-screw inerter is mainly supplied by angular kinetic energy of flywheel. *T* and *ω*_F_ are expressed as Eq. (2).

(2) $$T=\frac{1}{2}I_{F}\omega_{F}^{2},\omega_{F}=\frac{2\pi }{P_{b}}\left(v_{2}-v_{1}\right)$$

Based on Eq. (2), *T* is written in the form of Eq. (3).

(3) $$T=\frac{1}{2}I_{F}{\left(\frac{2\pi }{P_{b}}\right)}^{2}{\left(v_{2}-v_{1}\right)}^{2}$$

Based on Lagrange equation method, for the generalized coordinate *x*_2_, there are relationships expressed as Eq. (4) and Eq. (5).

(4) $$\frac{\partial T}{\partial v_{2}}=I_{F}{\left(\frac{2\pi }{P_{b}}\right)}^{2}\left(v_{2}-v_{1}\right)$$

(5) $$\frac{d}{dt}\left(\frac{\partial T}{\partial v_{2}}\right)=I_{F}{\left(\frac{2\pi }{P_{b}}\right)}^{2}\left({\overset{．}{v}}_{2}-{\overset{．}{v}}_{1}\right)=F_{2}$$

Based on Lagrange equation method, for the generalized coordinate *x*_1_, there are relationships expressed as Eq. (6) and Eq. (7).

(6) $$\frac{\partial T}{\partial v_{1}}=-I_{F}{\left(\frac{2\pi }{P_{b}}\right)}^{2}\left(v_{2}-v_{1}\right)$$

(7) $$\frac{d}{dt}\left(\frac{\partial T}{\partial v_{1}}\right)=-I_{F}{\left(\frac{2\pi }{P_{b}}\right)}^{2}\left({\overset{．}{v}}_{2}-{\overset{．}{v}}_{1}\right)=-F_{1}$$

Based on Eq. (5) and Eq. (7), forces *F*_2_ and *F*_1_ can be expressed as Eq. (8).

(8) $$F_{2}=F_{1}=I_{F}{\left(\frac{2\pi }{P_{b}}\right)}^{2}\left({\overset{．}{v}}_{2}-{\overset{．}{v}}_{1}\right)$$

Therefore, the dynamic relationship in a typical nut rotary type ball-screw inerter in Fig. 3 and a typical screw rotary type ball-screw inerter in Fig. 2, is expressed as Eq. (1).

The ball-screw inerter is compact and easy to be combined with electromechanical system. In the past two decades, different ball-screw inerters with mechanical structures and electromechanical structures have been proposed.

#### 2.2.2 Rack-and-pinion inerter

**Fig. 4:**
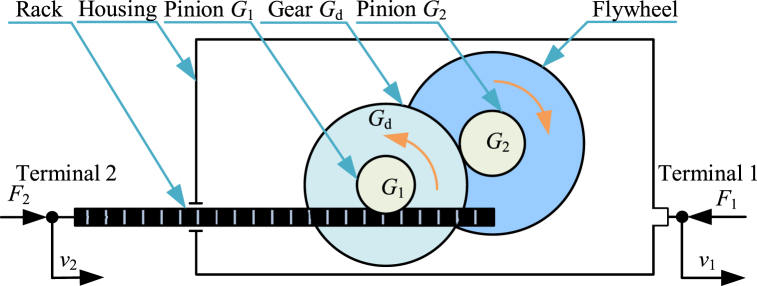
Diagram of a typical rack-and-pinion inerter [1].

Rack-and-pinion inerter [1,3,6,16,42,46,77,94,[131], [132], [133], [134], [135], [136], [137], [138], [139], [140]] is a kind of typical mechanical inerter and the early physical realization of inerter. The diagram of a typical rack-and-pinion inerter [1] is shown as Fig. 4. A typical rack-and-pinion inerter mainly consists of a pair of rack and pinion (rack and pinion *G*_1_), a pair of speed increaser gear and pinion (gear *G*_d_ and pinion *G*_2_), a flywheel as well as a housing. Housing is terminal 1, and rack is terminal 2. The two terminals can move horizontally relatively. The pinion *G*_1_ is fixed with the gear *G*_d_. The rack can drive the pinion *G*_1_ and gear *G*_d_ to rotate at the same angular velocity. The pinion *G*_2_ is fixed with the flywheel. And the gear *G*_d_ can drive the pinion *G*_2_ and the flywheel at an increased angular velocity. Angular speed of the pinion *G*_2_ is several times of the angular speed of the gear *G*_d_. Moment of inertia of flywheel is far more than moment of inertia of pinion *G*_2_. As a result, kinetic energy of a rack-and-pinion inerter is mainly supplied by angular kinetic energy of flywheel.

Only considering the angular kinetic energy of the flywheel, ignoring the friction and other factors, the dynamic relationship in a typical rack-and-pinion inerter in Fig. 4 is expressed as Eq. (9).

(9) $$\left\{ \begin{array}{c} F_{2}=F_{1}=I_{F}{\left(\frac{r_{d}}{r_{1}r_{2}}\right)}^{2}\left({\overset{．}{v}}_{2}-{\overset{．}{v}}_{1}\right)=m_{F}{\left(\gamma_{1}\gamma_{2}\right)}^{2}\left({\overset{．}{v}}_{2}-{\overset{．}{v}}_{1}\right)\\ b_{I}=m_{F}{\left(\gamma_{1}\gamma_{2}\right)}^{2},\gamma_{1}=\frac{r_{d}}{r_{1}},\gamma_{2}=\frac{r_{F}}{r_{2}} \end{array}\right.$$

where *F*_2_ is force applied to terminal 2 and *F*_1_ is force applied to terminal 1. *I*_F_ is moment of inertia of flywheel relative to its own rotation axis. *m*_F_ is mass of the flywheel. And *r*_1_ is pitch radius of pinion *G*_1_. *r*_d_ is pitch radius of gear *G*_d_. *r*_2_ is pitch radius of pinion *G*_2_. *r*_F_ is radius of gyration of flywheel. *γ*_1_ and *γ*_2_ are dimensionless amplification factors. *b*_I_ is the ideal inertance of inerter.

The normative derivation process based on Lagrange equation method of the dynamic relationships in a typical rack-and-pinion inerter is shown below. Kinetic energy of inerter is denoted as *T*. Kinetic energy of a rack-and-pinion inerter is mainly supplied by angular kinetic energy of flywheel. Angular velocity of flywheel is denoted as *ω*_F_. Kinetic energy of inerter *T* and angular velocity of flywheel *ω*_F_ are expressed as Eq. (10).

(10) $$T=\frac{1}{2}I_{F}\omega_{F}^{2},\omega_{F}=\frac{v_{2}-v_{1}}{r_{1}}\cdot \frac{r_{d}}{r_{2}}$$

Based on Eq. (10), *T* is written in the form of Eq. (11).

(11) $$T=\frac{1}{2}I_{F}{\left(\frac{r_{d}}{r_{1}r_{2}}\right)}^{2}{\left(v_{2}-v_{1}\right)}^{2}$$

Based on Lagrange equation method, for the generalized coordinate *x*_2_, there are relationships expressed as Eq. (12) and Eq. (13).

(12) $$\frac{\partial T}{\partial v_{2}}=I_{F}{\left(\frac{r_{d}}{r_{1}r_{2}}\right)}^{2}\left(v_{2}-v_{1}\right)$$

(13) $$\frac{d}{dt}\left(\frac{\partial T}{\partial v_{2}}\right)=I_{F}{\left(\frac{r_{d}}{r_{1}r_{2}}\right)}^{2}\left({\overset{．}{v}}_{2}-{\overset{．}{v}}_{1}\right)=F_{2}$$

Based on Lagrange equation method, for the generalized coordinate *x*_1_, there are relationships expressed as Eq. (14) and Eq. (15).

(14) $$\frac{\partial T}{\partial v_{1}}=-I_{F}{\left(\frac{r_{d}}{r_{1}r_{2}}\right)}^{2}\left(v_{2}-v_{1}\right)$$

(15) $$\frac{d}{dt}\left(\frac{\partial T}{\partial v_{1}}\right)=-I_{F}{\left(\frac{r_{d}}{r_{1}r_{2}}\right)}^{2}\left({\overset{．}{v}}_{2}-{\overset{．}{v}}_{1}\right)=-F_{1}$$

Based on Eq. (13) and Eq. (15), forces *F*_2_ and *F*_1_ can be expressed as Eq. (16).

(16) $$F_{2}=F_{1}=I_{F}{\left(\frac{r_{d}}{r_{1}r_{2}}\right)}^{2}\left({\overset{．}{v}}_{2}-{\overset{．}{v}}_{1}\right)$$

And the inertia *I*_F_ can be expressed as Eq. (17).

(17) $$I_{F}=m_{F}r_{F}^{2}$$

Therefore, the dynamic relationship in the typical rack-and-pinion inerter in Fig. 4 is expressed as Eq. (9).

Rack-and-pinion inerter in Fig. 4 is a typical single stage increaser rack-and-pinion inerter. And there are rack-and-pinion inerters with multi-increaser gears between rack-and-pinion and flywheel.

#### 2.2.3 Hydraulic inerter

**Fig. 5:**
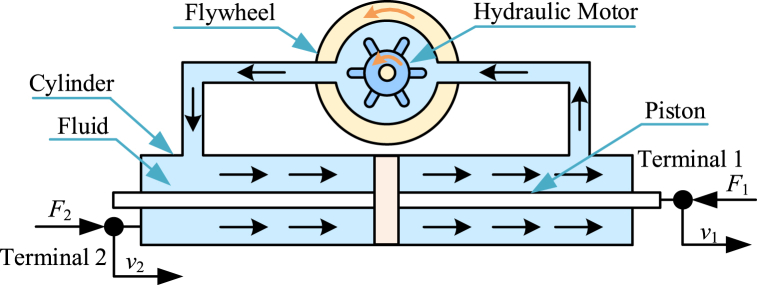
Diagram of a typical hydraulic inerter [160].

Hydraulic inerter [17,160,161] is another kind of typical mechanical inerter. Diagram of a typical hydraulic inerter [160] is shown as Fig. 5. A typical hydraulic inerter mainly consists of a hydraulic cylinder, hydraulic pipelines, a piston, a hydraulic motor and a flywheel. Hydraulic cylinder is terminal 2, and piston is terminal 1. Piston and hydraulic cylinder can move horizontally relatively. Flywheel is fixed to the hydraulic motor. Piston can drive hydraulic motor and flywheel to rotate at the same angular velocity, by driving the pressure fluid. Moment of inertia of flywheel is far more than moment of inertia of hydraulic motor's rotor. As a result, kinetic energy of a typical hydraulic inerter is mainly supplied by angular kinetic energy of flywheel.

Only considering the angular kinetic energy of the flywheel, ignoring the friction, pressure loss and other factors, the dynamic relationships in a typical hydraulic inerter in Fig. 5 can be expressed as Eq. (18).

(18) $$F_{2}=F_{1}=b_{I}\left({\overset{．}{v}}_{2}-{\overset{．}{v}}_{1}\right),b_{I}=I_{F}{\left(\frac{S_{p}}{Q_{m}}\right)}^{2}$$

where *S*_p_ is area of piston. *Q*_m_ is displacement of hydraulic motor.

Normative derivation process based on Lagrange equation method of the dynamic relationships in the typical hydraulic inerter is shown below. Kinetic energy of a typical hydraulic inerter is mainly supplied by the angular kinetic energy of flywheel. *T* and *ω*_F_ are expressed as Eq. (19).

(19) $$T=\frac{1}{2}I_{F}\omega_{F}^{2},\omega_{F}=\frac{S_{p}}{Q_{m}}\left(v_{2}-v_{1}\right)$$

Based on Eq. (19), *T* is written in the form of Eq. (20).

(20) $$T=\frac{1}{2}I_{F}{\left(\frac{S_{p}}{Q_{m}}\right)}^{2}{\left(v_{2}-v_{1}\right)}^{2}$$

Based on Lagrange equation method, for the generalized coordinate *x*_2_, there are relationships expressed as Eq. (21) and Eq. (22).

(21) $$\frac{\partial T}{\partial v_{2}}=I_{F}{\left(\frac{S_{p}}{Q_{m}}\right)}^{2}\left(v_{2}-v_{1}\right)$$

(22) $$\frac{d}{dt}\left(\frac{\partial T}{\partial v_{2}}\right)=I_{F}{\left(\frac{S_{p}}{Q_{m}}\right)}^{2}\left({\overset{．}{v}}_{2}-{\overset{．}{v}}_{1}\right)=F_{2}$$

Based on Lagrange equation method, for the generalized coordinate *x*_1_, there are relationships expressed as Eq. (23) and Eq. (24).

(23) $$\frac{\partial T}{\partial v_{1}}=-I_{F}{\left(\frac{S_{p}}{Q_{m}}\right)}^{2}\left(v_{2}-v_{1}\right)$$

(24) $$\frac{d}{dt}\left(\frac{\partial T}{\partial v_{1}}\right)=-I_{F}{\left(\frac{S_{p}}{Q_{m}}\right)}^{2}\left({\overset{．}{v}}_{2}-{\overset{．}{v}}_{1}\right)=-F_{1}$$

Based on Eq. (22) and Eq. (24), the forces *F*_2_ and *F*_1_ can be expressed as Eq. (25).

(25) $$F_{2}=F_{1}=I_{F}{\left(\frac{S_{p}}{Q_{m}}\right)}^{2}\left({\overset{．}{v}}_{2}-{\overset{．}{v}}_{1}\right)$$

Therefore, the dynamic relationships in a typical hydraulic inerter in Fig. 5 can be expressed as Eq. (18).

Actually, the dynamic characteristics of the hydraulic inerter is influenced by many factors, such as pressure loss, compression effect and friction, etc.

#### 2.2.4 Fluid inerter

**Fig. 6:**
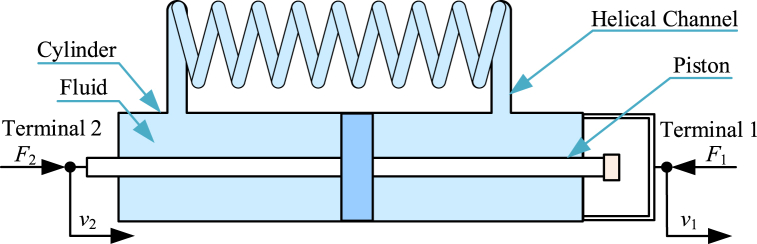
Diagram of a typical fluid inerter [162].

Fluid inerter [20,23,48,54,[162], [163], [164], [165], [166], [167], [168], [169]] is another kind of typical mechanical inerter. The diagram of a typical fluid inerter [162] is shown as Fig. 6. A typical fluid inerter mainly consists of a hydraulic cylinder, hydraulic pipelines, helical channel, a piston. Piston is terminal 2, and hydraulic cylinder is terminal 1. Piston and hydraulic cylinder can move horizontally relatively. Piston can drive fluid to flow through helical channel. Area of piston is far more than cross sectional area of helical channel. And velocity of fluid in helical channel is far more than that in hydraulic cylinder. It can be validated that kinetic energy of a fluid inerter is mainly supplied by fluid in helical channel.

Only considering kinetic energy of fluid in helical channel, ignoring friction, the parasitic damping and other factors, the dynamic relationships in a typical fluid inerter in Fig. 6 can be expressed as Eq. (26).

(26) $$F_{2}=F_{1}=b_{I}\left({\overset{．}{v}}_{2}-{\overset{．}{v}}_{1}\right),b_{I}=\rho l_{f}\left(\frac{S_{1}^{2}}{S_{2}}\right)$$

where *l*_f_ is length of helical channel and *ρ* is density of fluid. *S*_1_ is area of piston and *S*_2_ is cross sectional area of helical channel.

Normative derivation process based on Lagrange equation method of dynamic relationships in a typical fluid inerter is shown below. Kinetic energy of a fluid inerter is mainly supplied by fluid in helical channel. *T* is expressed as Eq. (27).

(27) $$T=\frac{1}{2}\rho S_{2}l_{f}u^{2},u=\frac{S_{1}}{S_{2}}\left(v_{2}-v_{1}\right)$$

where *u* is velocity of fluid in helical channel.

Based on Eq. (27), *T* is written in the form of Eq. (28).

(28) $$T=\frac{1}{2}\rho S_{2}l_{f}{\left(\frac{S_{1}}{S_{2}}\right)}^{2}{\left(v_{2}-v_{1}\right)}^{2}=\frac{1}{2}\rho l_{f}\left(\frac{S_{1}^{2}}{S_{2}}\right){\left(v_{2}-v_{1}\right)}^{2}$$

Based on Lagrange equation method, for the generalized coordinate *x*_2_, there are relationships expressed as Eq. (29) and Eq. (30).

(29) $$\frac{\partial T}{\partial v_{2}}=\rho l_{f}\left(\frac{S_{1}^{2}}{S_{2}}\right)\left(v_{2}-v_{1}\right)$$

(30) $$\frac{d}{dt}\left(\frac{\partial T}{\partial v_{2}}\right)=\rho l_{f}\left(\frac{S_{1}^{2}}{S_{2}}\right)\left({\overset{．}{v}}_{2}-{\overset{．}{v}}_{1}\right)=F_{2}$$

Based on Lagrange equation method, for the generalized coordinate *x*_1_, there are relationships expressed as Eq. (31) and Eq. (32).

(31) $$\frac{\partial T}{\partial v_{1}}=-\rho l_{f}\left(\frac{S_{1}^{2}}{S_{2}}\right)\left(v_{2}-v_{1}\right)$$

(32) $$\frac{d}{dt}\left(\frac{\partial T}{\partial v_{1}}\right)=-\rho l_{f}\left(\frac{S_{1}^{2}}{S_{2}}\right)\left({\overset{．}{v}}_{2}-{\overset{．}{v}}_{1}\right)=-F_{1}$$

Based on Eq. (30) and Eq. (32), the forces *F*_2_ and *F*_1_ can be expressed as Eq. (33).

(33) $$F_{2}=F_{1}=\rho l_{f}\left(\frac{S_{1}^{2}}{S_{2}}\right)\left({\overset{．}{v}}_{2}-{\overset{．}{v}}_{1}\right)$$

Therefore, dynamic relationship in a typical fluid inerter in Fig. 6 is expressed as Eq. (26).

In physical fluid inerter, the influence of parasitic damping is almost inevitable. Modeling and utilization of parasitic damping of fluid inerter have been focused by researchers.

#### 2.2.5 Living-hinge inerter

**Fig. 7:**
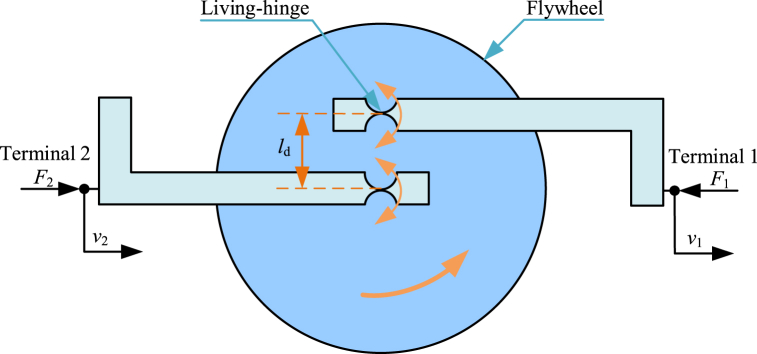
Diagram of a typical living-hinge inerter [170].

Living-hinge inerter [[170], [171], [172]] is a novel type of mechanical inerter proposed in recent years. The physical realization of a living-hinge inerter is quite different from other mechanical inerters. Diagram of a typical living-hinge inerter [170] is shown as Fig. 7. A typical living-hinge inerter mainly consists of two living-hinges, a flywheel as well as two vertical connecting rods. The left vertical connecting rod is the terminal 2, and the right vertical connecting rod is the terminal 1. The living-hinge can be twisted and deformed, equivalent to a revolute pair. The center of gyration of one of the two living-hinges is at the center of flywheel, and the center of gyration of the other living-hinge deviates from the center of flywheel. Two vertical connecting rods can move horizontally relatively, and drive flywheel to rotate. Kinetic energy of a typical living-hinge inerter is mainly supplied by angular kinetic energy of flywheel.

In the case of a small rotation angle of flywheel, only considering angular kinetic energy of flywheel, dynamic relationship in a typical living-hinge inerter in Fig. 7 is expressed as Eq. (34).

(34) $$F_{2}=F_{1}=b_{I}\left({\overset{．}{v}}_{2}-{\overset{．}{v}}_{1}\right),b_{I}=I_{F}\left(\frac{1}{l_{d}^{2}}\right)$$

where *l*_d_ is distance between the two living-hinges’ centers of gyration.

Normative derivation process based on Lagrange equation method of dynamic relationships in a typical living-hinge inerter is shown below. Kinetic energy of a typical living-hinge inerter is mainly supplied by angular kinetic energy of flywheel. *T* and *ω*_F_ are expressed as Eq. (35).

(35) $$T=\frac{1}{2}I_{F}\omega_{F}^{2},\omega_{F}=\frac{v_{2}-v_{1}}{l_{d}}$$

Based on Eq. (35), *T* is written in the form of Eq. (36).

(36) $$T=\frac{1}{2}I_{F}\frac{1}{l_{d}^{2}}{\left(v_{2}-v_{1}\right)}^{2}$$

Based on Lagrange equation method, for the generalized coordinate *x*_2_, there are relationships expressed as Eq. (37) and Eq. (38).

(37) $$\frac{\partial T}{\partial v_{2}}=I_{F}\frac{1}{l_{d}^{2}}\left(v_{2}-v_{1}\right)$$

(38) $$\frac{d}{dt}\left(\frac{\partial T}{\partial v_{2}}\right)=I_{F}\frac{1}{l_{d}^{2}}\left({\overset{．}{v}}_{2}-{\overset{．}{v}}_{1}\right)=F_{2}$$

Based on Lagrange equation method, for the generalized coordinate *x*_1_, there are relationships expressed as Eq. (39) and Eq. (40).

(39) $$\frac{\partial T}{\partial v_{1}}=-I_{F}\frac{1}{l_{d}^{2}}\left(v_{2}-v_{1}\right)$$

(40) $$\frac{d}{dt}\left(\frac{\partial T}{\partial v_{1}}\right)=-I_{F}\frac{1}{l_{d}^{2}}\left({\overset{．}{v}}_{2}-{\overset{．}{v}}_{1}\right)=-F_{1}$$

Based on Eq. (38) and Eq. (40), the forces *F*_2_ and *F*_1_ can be expressed as Eq. (41).

(41) $$F_{2}=F_{1}=I_{F}\frac{1}{l_{d}^{2}}\left({\overset{．}{v}}_{2}-{\overset{．}{v}}_{1}\right)$$

Therefore, dynamic relationship in a typical living-hinge inerter in Fig. 7 is expressed as Eq. (34).

The living-hinge inerter is almost frictionless, with small additional damping. Currently, the living-hinge inerter is small-size and used as inerter-based dynamic vibration absorbers.

#### 2.2.6 Electromechanical inerter

Electromechanical inerter [11,26,31,34,38,39,43,69,80,95,107,109,113,156,[173], [174], [175], [176], [177], [178], [179], [180], [181], [182], [183], [184], [185]] is the combination of the mechanical inerter and electrical system. There are two kinds of basic realization structures of the electromechanical inerters, i.e. the electromagnetic inerter and the hydraulic electric inerter.

##### 2.2.6.1 Electromagnetic inerter

**Fig. 8:**
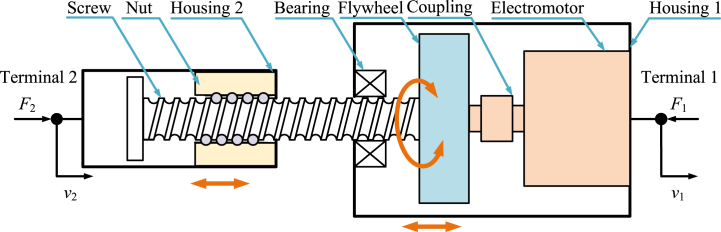
Diagram of a typical electromagnetic inerter [11].

Electromagnetic inerter [11,31,34,38,39,43,69,80,95,107,109,113,156,[173], [174], [175], [176], [177], [178],[181], [182], [183], [184], [185]] is the combination of a ball-screw inerter or a rack-and-pinion inerter and electrical system, commonly. Diagram of a typical electromagnetic inerter [11] is shown as Fig. 8. A typical electromagnetic inerter mainly consists of a pair of ball nut and screw, a flywheel, bearings, a coupling, two housings, an electromotor and its necessary circuit. Housing 2 is terminal 2, and housing 1 is terminal 1. Housing 2 and housing 1 can move horizontally relatively. Nut is fixed to housing 2. Flywheel is fixed to screw. Flywheel and rotor of electromotor are connected by coupling. Nut can drive screw, flywheel and rotor of electromotor to rotate at the same angular velocity.

Dynamic characteristics of an electromagnetic inerter is more complex than the mechanical inerter. The electromotor can be replaced by other mechanical-electrical conversion devices. And different electrical networks with resistors, capacitors and inductors can be installed in the electromotor's necessary circuit.

##### 2.2.6.2 Hydraulic electric inerter

**Fig. 9:**
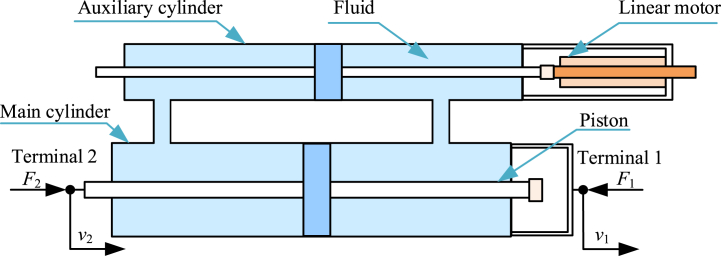
Diagram of a typical hydraulic electric inerter [180].

Hydraulic electric inerter [26,179,180] is the combination of a hydraulic inerter or a fluid inerter and electrical system, commonly. Diagram of a typical hydraulic electric inerter [180] is shown as Fig. 9. A typical hydraulic electric inerter mainly consists of a main hydraulic cylinder, pistons, an auxiliary cylinder, a linear motor and its necessary circuit, hydraulic pipelines and adapting pieces. The piston in main cylinder is terminal 2, and main hydraulic cylinder is terminal 1. Each piston and each hydraulic cylinder can move horizontally relatively. Moving rod of linear motor is fixed to piston in auxiliary cylinder. Piston in main cylinder can drive piston in auxiliary cylinder and moving rod of linear motor, by driving pressure fluid. Area of piston in main cylinder is more than area of piston in auxiliary cylinder. Velocity of the fluid in the auxiliary cylinder is more than that in the main cylinder.

Also, dynamic characteristics of a hydraulic electric inerter is more complex than the mechanical inerter. The linear motor can be replaced by other mechanical-electrical conversion devices. Different electrical networks with resistors, capacitors and inductors can be installed in the linear motor's necessary circuit.

#### 2.2.7 Novel nonlinear inerter

**Fig. 10:**
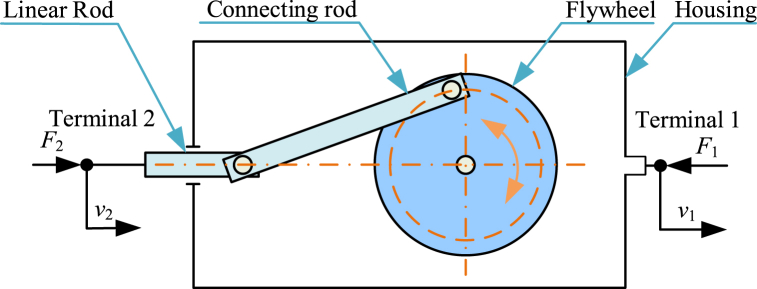
Diagram of a crank inerter [186].

Recently, some new inerters have been developed, such as crank inerters [[186], [187], [188]] and yoke-type nonlinear inerters [189], etc. Diagram of a crank inerter is shown in Fig. 10. Crank inerter [186] mainly consists of a linear rod, a connecting rod, a flywheel, a housing and other connections. The linear rod is terminal 2 and the housing is terminal 1. The linear rod and the housing can move horizontally relatively. The linear rod can drive the flywheel to rotate via the connecting rod. The crank inerter can provide variable inertance and negative stiffness. And vibration control systems with crank inerter are able to improve structural performances, containing frequency band and peak force transmissibility.

**Fig. 11:**
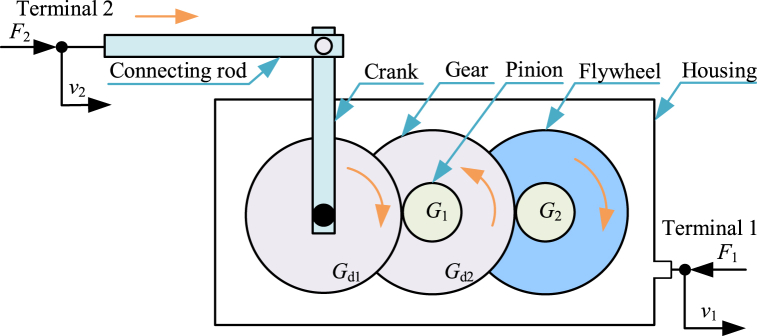
Diagram of a crank train inerter [187,188].

Similar with the crank inerter in Ref. [186], diagram of a crank train inerter [187,188] is shown in Fig. 11. Crank train inerter mainly consists of a connecting rod, a crank, gears and pinions, a flywheel and a housing. The connecting rod is terminal 2 and the housing is terminal 1. The connecting rod can drive the gear *G*_d1_ to rotate via the crank. The pinion *G*_1_ is fixed with the gear *G*_d2_. And the gear *G*_d1_ can drive the pinion *G*_1_ and the gear *G*_d2_ at an increased angular velocity. The pinion *G*_2_ is fixed with the flywheel. And the gear *G*_d2_ can drive the pinion *G*_2_ and the flywheel at an increased angular velocity. The crank train inerter can also provide variable inertance and negative stiffness. Under certain constraints, crank train inerter can be approximatively linearized.

**Fig. 12:**
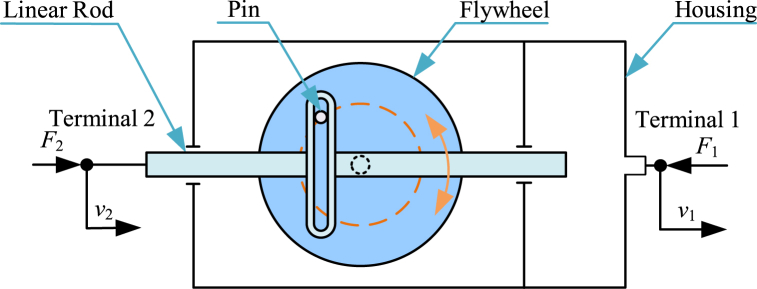
Diagram of a yoke-type nonlinear inerter [189].

Diagram of a yoke-type nonlinear inerter [189] is shown in Fig. 12. Yoke-type nonlinear inerter mainly consists of a linear rod, a pin, a flywheel, a housing and other connections. The linear rod is terminal 2 and the housing is terminal 1. The linear rod and the housing can move horizontally relatively. The pin is fixed with the flywheel. The linear rod can drive the flywheel to rotate via the pin. The yoke-type nonlinear inerter can also provide variable inertance and negative stiffness.

Synthesize different physical realizations of inerter, and a comparison of their characteristics is made in Table 1.

**Table 1:** Comparison of different physical realizations of inerter.

Type	Characteristics
Ball-screw inerter	●Large inertial amplification effect ●Back clearance between ball and screw ●Strong universality
Rack-and-pinion inerter	●Large inertial amplification effect ●Gear back-lash ●High loading capacity
Hydraulic inerter	●Strong nonlinearity and damping effect ●High loading capacity ●High sealing requirement
Fluid inerter	●Strong nonlinearity and damping effect ●High loading capacity ●High sealing requirement ●Easy installation
Living-hinge inerter	●Balanced forces ●Less friction ●Low loading capacity
Electromechanical inerter	●Multiple dynamic characteristics ●Complicated structure ●Research hotspot
Crank inerter	●Nonlinear inertance ●Less friction ●High loading capacity
Yoke-type nonlinear inerter	●Nonlinear inertance ●High loading capacity

## 3 Inerter-based vibration control

Compared with the traditional solutions, inerter has provided different routes to the vibration control problems of mechanical systems. Inerters have been proved to be effective in many applications. Three categories of common inerter-based vibration control systems: the energy dissipators based on inerters, the dynamic vibration absorbers based on inerters and the vibration isolators based on inerters, are explained in this section.

### 3.1 Energy dissipators based on inerters

**Fig. 13:**
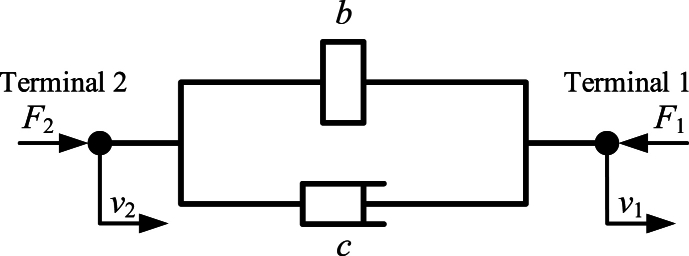
Equivalent network of the typical energy dissipator based on inerter [142].

Energy dissipators based on inerters [57,73,78,80,103,109,142,149,153,156,158,[191], [192], [193], [194], [195], [196], [197], [198], [199], [200]] is a kind of common inerter-based vibration control systems. Inerters themselves do not dissipate energy. When connected in parallel with damping elements, they can enhance the energy dissipation capability of the damping elements due to their displacement amplification ability. Additionally, there are many inerter-based energy dissipators, such as inertial mass dampers (IMDs) [80,103,109,181,201,202] and tuned viscous mass dampers (TVMDs) [57,142,[198], [199], [200],^203^]. Energy dissipators based on inerters are mainly applied in structure vibration control system of buildings and vibration control system of bridges. The equivalent network of the typical energy dissipator based on inerter [142] is shown in Fig. 13. The fundamental structure of the energy dissipators based on inerters is a structure with an inerter and a damper in parallel. The physical energy dissipators based on inerters can obtain large inertial force and viscous damping force, only with small mass and low viscosity of the equipment. And energy dissipation of energy dissipators based on inerter is improved by the speed amplifying mechanism of the inerter.

**Fig. 14:**
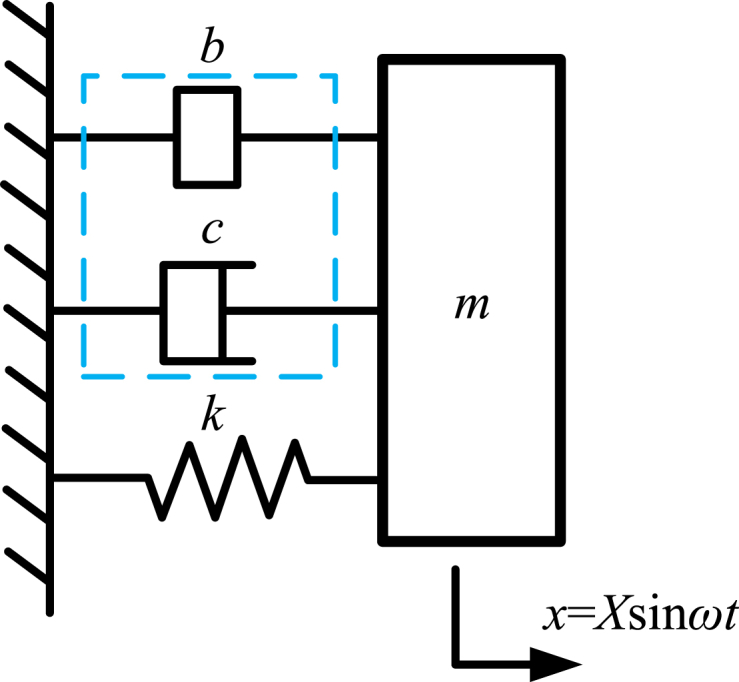
A typical application of energy dissipator based on inerter.

To explain the dynamic behavior of the typical energy dissipator based on inerter, a typical application of energy dissipator based on inerter is shown as Fig. 14. Mass of mass block is denoted as *m*. Displacement of mass block is denoted as *x*. The mass block moves in simple harmonic motion. And, displacement *x* is expressed as “*X*sin*ωt*”. *X* is amplitude of displacement *x* and *ω* is angular frequency of displacement *x*. The velocity of mass block is *v*. And the velocity *v* can be expressed as “*Xω*cos*ωt*”.

The inertial force of inerter *F*_b_ and damping force of damper *F*_c_ is expressed as Eq. (42).

(42) $$\left\{ \begin{array}{c} F_{b}=-bX\omega^{2}\;\sin\;\omega t\\ F_{c}=cX\omega \;\cos\;\omega t \end{array}\right.$$

**Fig. 15:**
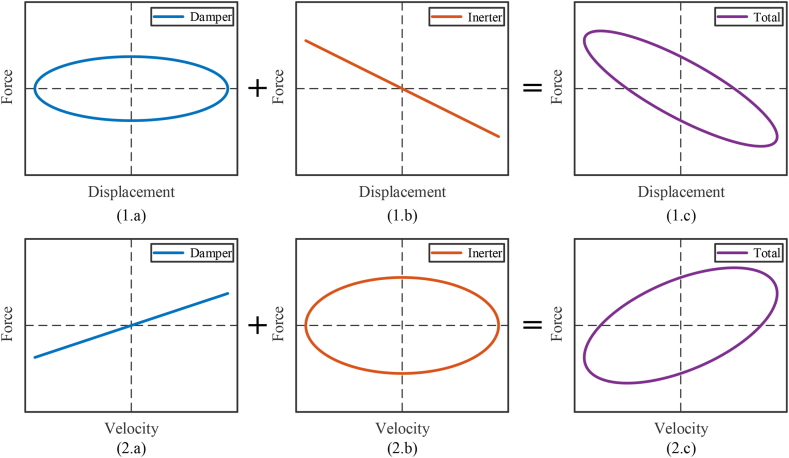
Dynamic behavior of the typical application of energy dissipator based on inerter.

The relationship of damping force of damper *F*_c_ and displacement *x* is expressed as Eq. (43). The relationship means an elliptical curve and is shown in Fig. 15(1.a).

(43) $$\frac{x^{2}}{X^{2}}+\frac{F_{c}^{2}}{{\left(cX\omega \right)}^{2}}=1$$

The relationship of inertial force of inerter *F*_b_ and displacement *x* is expressed as Eq. (44). And Eq. (44) means negative stiffness of the inerter. And the relationship is shown as Fig. 15(1.b).

(44) $$\frac{F_{b}}{x}=-b\omega^{2}$$

The relationship of damping force of damper *F*_c_ and velocity *v* is expressed as Eq. (45). And the relationship is shown as Fig. 15(2.a).

(45) $$\frac{F_{c}}{v}=c$$

The relationship of inertial force of inerter *F*_b_ and velocity *v* is expressed as Eq. (46). The relationship means an elliptical curve and is shown as Fig. 15(2.b).

(46) $$\frac{v^{2}}{{\left(X\omega \right)}^{2}}+\frac{F_{b}^{2}}{{\left(bX\omega^{2}\right)}^{2}}=1$$

The dynamic behavior of a typical application of energy dissipator based on inerter is shown in Fig. 15. As mentioned above, Fig. 15(1.a) shows relationship between damping force of damper *F*_c_ and displacement *x*. Fig. 15(1.b) shows relationship of inertial force of inerter *F*_b_ and displacement *x*. Fig. 15(1.c) shows relationship of the total force *F*_c_ + *F*_b_ and displacement *x*. Fig. 15(2.a) shows relationship of damping force of damper *F*_c_ and velocity *v*. Fig. 15(2.b) shows relationship of inertial force of inerter *F*_b_ and velocity *v*. Fig. 15(2.c) shows relationship of the total force *F*_c_ + *F*_b_ and velocity *v*. Fig. 15(1.c) and Fig. 15(2.c) are used to show the characteristics of the energy dissipators based on inerters.

As the inerter devices should be two-terminal connected, the installation on slender structures, such as practical tall structures, for vibration control is difficult. Several means to address the realization of inerter-based vibration control are provided. It can be connected between layers to investigate location dependence [204]. Alternatively, inerter-based double tuned mass dampers are provided and investigated to achieve lightweight vibration control [205]. The inerter-based damping system can also be installed with cable supported structures [206].

The physical energy dissipators based on inerters mainly adopt ball-screw inerters and rack-and-pinion inerters. Ball-screw inerter is compact and easy to be combined with viscous damper and electromechanical system. Also, the electromagnetic damper has been applied in the energy dissipators based on inerters. And there are energy dissipators based on inerters with a spring connected in series, to obtain larger dashpot deformation and better energy dissipation.

### 3.2 Dynamic vibration absorbers based on inerters

**Fig. 16:**
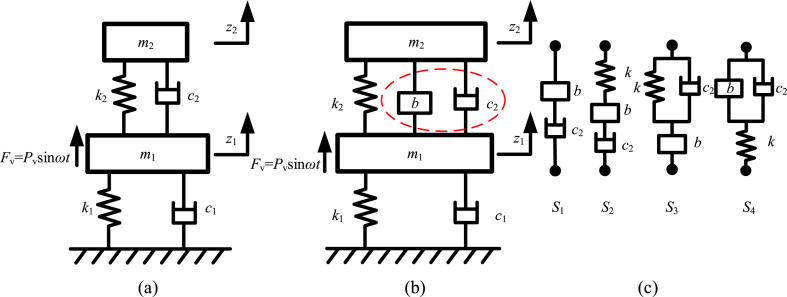
A traditional dynamic vibration absorber as well as dynamic vibration absorbers based on inerters [208].

The dynamic vibration absorbers based on inerters [49,67,81,111,144,158,172,175,176,[207], [208], [209], [210], [211], [212], [213], [214], [215], [216], [217], [218], [219], [220], [221], [222], [223], [224], [225], [226], [227], [228], [229], [230], [231]] is another kind of common inerter-based vibration control systems. Dynamic vibration absorber is an auxiliary inertial system, which is attached to a primary vibration system, to reduce or control vibration of primary system. And dynamic vibration absorbers based on inerters are mainly applied in structure vibration control system of buildings, vibration control system of bridges, vibration control system of rotating machines, vibration energy harvesting devices, linear and nonlinear energy sink, etc. The traditional dynamic vibration absorber can only use the one-terminal mass to compose the auxiliary inertial system. Dynamic vibration absorber based on inerter is different, the two-terminal inerter is used to obtain required control performance. And the structures of dynamic vibration absorbers based on inerters are diverse, with categories of applications. A traditional dynamic vibration absorber as well as dynamic vibration absorbers based on inerters [208] are shown as Fig. 16.

Primary vibration system contains the mass *m*_1_, the spring *k*_1_ and the damper *c*_1_. Fig. 16(a) shows traditional dynamic vibration absorber, which contains the auxiliary mass *m*_2_, the spring *k*_2_ and the damper *c*_2_. Fig. 16(b) shows a dynamic vibration absorber based on inerter [208], which contains the auxiliary mass *m*_2_, the auxiliary inerter *b*, the spring *k*_2_ and the damper *c*_2_. Fig. 16(c) shows four kinds of structures used in other dynamic vibration absorbers based on inerters, which can replace the inerter *b* and the damper *c*_2_ noted by red dashed ellipse in Fig. 16(b). And the structure *S*_4_ in Fig. 16(c) is tuned viscous mass damper (TVMD) [57,142,[198], [199], [200],^203^]. Displacement of mass *m*_1_ is denoted as *z*_1_, and displacement of auxiliary mass *m*_2_ is denoted as *z*_2_. The simple harmonic exciting force applied to the mass *m*_1_ is denoted as *F*_v_. And, the exciting force *F*_v_ can be expressed as “*P*_v_sin*ωt*”. *P*_v_ is amplitude of exciting force *F*_v_. *ω* is angular frequency of exciting force *F*_v_. Vibration of primary vibration system, i.e. the displacement *z*_1_, is mainly concerned.

The dynamic relationships in the vibration system with a traditional dynamic vibration absorber in Fig. 16(a) are expressed as Eq. (47).

(47) $$\left[\begin{array}{cc} m_{1} & 0\\ 0 & m_{2} \end{array}\right]\left[\begin{array}{c} \overset{‥\;}{z_{1}}\\ \overset{‥\;}{z_{2}} \end{array}\right]+\left[\begin{array}{cc} k_{1}+k_{2} & -k_{2}\\ -k_{2} & k_{2} \end{array}\right]\left[\begin{array}{c} z_{1}\\ z_{2} \end{array}\right]+\left[\begin{array}{cc} c_{1}+c_{2} & -c_{2}\\ -c_{2} & c_{2} \end{array}\right]\left[\begin{array}{c} \overset{.\;}{z_{1}}\\ \overset{.\;}{z_{2}} \end{array}\right]=\left[\begin{array}{c} P_{v}\;\sin\;\omega t\\ 0 \end{array}\right]$$

The dynamic relationships in the vibration system with dynamic vibration absorber based on inerter in Fig. 16(b) are expressed as Eq. (48).

(48) $$\left[\begin{array}{cc} m_{1}+b & -b\\ -b & m_{2}+b \end{array}\right]\left[\begin{array}{c} \overset{‥\;}{z_{1}}\\ \overset{‥\;}{z_{2}} \end{array}\right]+\left[\begin{array}{cc} k_{1}+k_{2} & -k_{2}\\ -k_{2} & k_{2} \end{array}\right]\left[\begin{array}{c} z_{1}\\ z_{2} \end{array}\right]+\left[\begin{array}{cc} c_{1}+c_{2} & -c_{2}\\ -c_{2} & c_{2} \end{array}\right]\left[\begin{array}{c} \overset{.\;}{z_{1}}\\ \overset{.\;}{z_{2}} \end{array}\right]=\left[\begin{array}{c} P_{v}\;\sin\;\omega t\\ 0 \end{array}\right]$$

It is obvious that Eq. (47) is the situation when the *b* = 0 in Eq. (48). Considering the steady-state solution of Eq. (48), the simple harmonic exciting force *F*_v_ can be expressed as “*P*_v_e^i*ωt*^”. And complex amplitudes of *z*_1_ and *z*_2_ are denoted as ${\bar{A}}_{1}$ and ${\bar{A}}_{2}$, respectively. Complex amplitudes of *z*_1_ and *z*_2_ can be expressed as Eq. (49).

(49) $$\left[\begin{array}{c} z_{1}\\ z_{2} \end{array}\right]=\left[\begin{array}{c} {\bar{A}}_{1}\\ {\bar{A}}_{2} \end{array}\right]e^{i\omega t}$$

Based on Eq. (49) and Eq. (48), complex amplitudes of *z*_1_ and *z*_2_ can be obtained and expressed as Eq. (50).

(50) $$\left[\begin{array}{c} {\bar{A}}_{1}\\ {\bar{A}}_{2} \end{array}\right]={\left[\begin{array}{cc} k_{1}+k_{2}‐\left(m_{1}+b\right)\omega^{2}+i\left(c_{1}+c_{2}\right)\omega & ‐\left(k_{2}‐b\omega^{2}+ic_{2}\omega \right)\\ ‐\left(k_{2}‐b\omega^{2}+ic_{2}\omega \right) & k_{2}‐\left(m_{2}+b\right)\omega^{2}+ic_{2}\omega \end{array}\right]}^{‐1}\left[\begin{array}{c} P_{v}\\ 0 \end{array}\right]=\frac{P_{v}}{\Delta \left(\omega \right)}\left[\begin{array}{c} k_{2}‐\left(m_{2}+b\right)\omega^{2}+ic_{2}\omega \\ k_{2}‐b\omega^{2}+ic_{2}\omega \end{array}\right]$$

Δ(*ω*) can be expressed as Eq. (51).

(51) $$\Delta \left(\omega \right)=\left(k_{1}+k_{2}‐m_{1}\omega^{2}‐b\omega^{2}+ic_{1}\omega +ic_{2}\omega \right)\left(k_{2}‐m_{2}\omega^{2}‐b\omega^{2}+ic_{2}\omega \right)‐{\left(k_{2}‐b\omega^{2}+ic_{2}\omega \right)}^{2}=\left(m_{1}m_{2}+m_{1}b+m_{2}b\right)\omega^{4}‐\left(k_{2}m_{1}+k_{2}m_{2}+k_{1}m_{2}+k_{1}b+c_{1}c_{2}\right)\omega^{2}+k_{1}k_{2}+i\left(k_{2}c_{1}+k_{1}c_{2}\right)\omega ‐i\left(m_{1}c_{2}+m_{2}c_{2}+m_{2}c_{1}+bc_{1}\right)\omega^{3}$$

Therefore, the complex amplitude of *z*_1_ can be expressed as Eq. (52).

(52) $${\bar{A}}_{1}=\frac{P_{v}\left(k_{2}-m_{2}\omega^{2}-b\omega^{2}+ic_{2}\omega \right)}{\left(m_{1}m_{2}+m_{1}b+m_{2}b\right)\omega^{4}-\left(k_{2}m_{1}+k_{2}m_{2}+k_{1}m_{2}+k_{1}b+c_{1}c_{2}\right)\omega^{2}+k_{1}k_{2}+i\left(k_{2}c_{1}+k_{1}c_{2}\right)\omega -i\left(m_{1}c_{2}+m_{2}c_{2}+m_{2}c_{1}+bc_{1}\right)\omega^{3}}$$

Furthermore, the modulus of the complex amplitude of *z*_1_ is *A*_1_ and can be expressed as Eq. (53).

(53) $$A_{1}=\frac{P_{v}\sqrt{{\left(k_{2}‐m_{2}\omega^{2}‐b\omega^{2}\right)}^{2}+{\left(c_{2}\omega \right)}^{2}}}{\sqrt{{\left[\left(m_{1}m_{2}+m_{1}b+m_{2}b\right)\omega^{4}‐\left(k_{2}m_{1}+k_{2}m_{2}+k_{1}m_{2}+k_{1}b+c_{1}c_{2}\right)\omega^{2}+k_{1}k_{2}\right]}^{2}+{\left[\left(k_{2}c_{1}+k_{1}c_{2}\right)\omega ‐\left(m_{1}c_{2}+m_{2}c_{2}+m_{2}c_{1}+bc_{1}\right)\omega^{3}\right]}^{2}}}$$

Natural frequency of primary vibration system without dynamic vibration absorber is *ω*_0_, expressed as Eq. (54). The frequency ratio can be expressed as “*ω*/*ω*_0_”.

(54) $$\omega_{0}=\sqrt{\frac{k_{1}}{m_{1}}}$$

The mechanical admittance's modular form of displacement of primary mass *z*_1_ and exciting force *F*_v_ is *Y*_m_, and is expressed as Eq. (55).

(55) $$Y_{m}=\frac{A_{1}}{P_{v}}=\frac{\sqrt{{\left(k_{2}‐m_{2}\omega^{2}‐b\omega^{2}\right)}^{2}+{\left(c_{2}\omega \right)}^{2}}}{\sqrt{{\left[\left(m_{1}m_{2}+m_{1}b+m_{2}b\right)\omega^{4}‐\left(k_{2}m_{1}+k_{2}m_{2}+k_{1}m_{2}+k_{1}b+c_{1}c_{2}\right)\omega^{2}+k_{1}k_{2}\right]}^{2}+{\left[\left(k_{2}c_{1}+k_{1}c_{2}\right)\omega ‐\left(m_{1}c_{2}+m_{2}c_{2}+m_{2}c_{1}+bc_{1}\right)\omega^{3}\right]}^{2}}}$$

Considering an undamped case: *c*_1_ = 0, *c*_2_ = 0, solve the function “Δ(*ω*) = 0”, the two natural frequencies of vibration system in Fig. 16(b), *ω*_n1_ and *ω*_n2_ can be obtained and expressed as Eq. (56).

(56) $$\left\{ \begin{array}{c} \omega_{n1}=\sqrt{\frac{\left(k_{2}m_{1}+k_{2}m_{2}+k_{1}m_{2}+k_{1}b\right)-\sqrt{{\left(k_{2}m_{1}+k_{2}m_{2}+k_{1}m_{2}+k_{1}b\right)}^{2}-4\left(m_{1}m_{2}+m_{1}b+m_{2}b\right)k_{1}k_{2}}}{2\left(m_{1}m_{2}+m_{1}b+m_{2}b\right)}}\\ \omega_{n2}=\sqrt{\frac{\left(k_{2}m_{1}+k_{2}m_{2}+k_{1}m_{2}+k_{1}b\right)+\sqrt{{\left(k_{2}m_{1}+k_{2}m_{2}+k_{1}m_{2}+k_{1}b\right)}^{2}-4\left(m_{1}m_{2}+m_{1}b+m_{2}b\right)k_{1}k_{2}}}{2\left(m_{1}m_{2}+m_{1}b+m_{2}b\right)}} \end{array}\right.$$

**Fig. 17:**
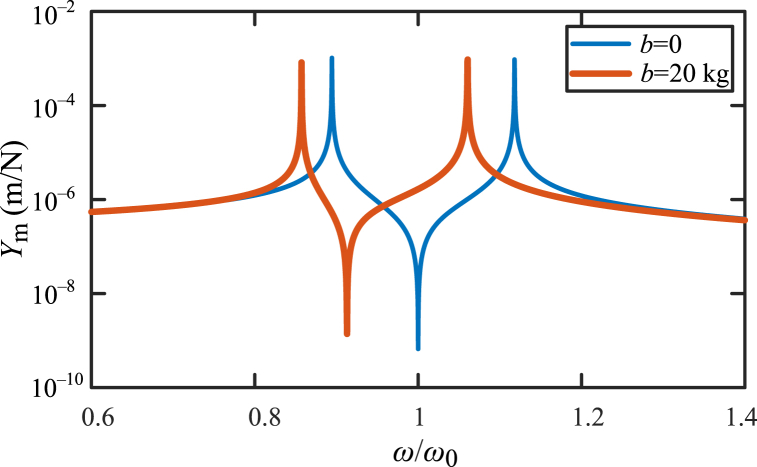
Dynamic behavior of a typical application of a dynamic vibration absorber based on inerter.

Considering the undamped case, and the parameters are that: *m*_1_ = 2000 kg, *m*_2_ = 100 kg, *b* = 20 kg, *k*_1_ = 3 × 10^6^ N/m, *k*_2_ = 1.5 × 10^5^ N/m, *c*_1_ = 0, *c*_2_ = 0. Curves of mechanical admittance *Y*_m_ versus the frequency ratio *ω*/*ω*_0_ in the vibration systems shown in Fig. 16(a) and (b), are shown as Fig. 17. Thin blue line is the mechanical admittance *Y*_m_ of the vibration system with traditional dynamic vibration absorber in Fig. 16(a). Thick red line is the mechanical admittance *Y*_m_ of vibration system with a dynamic vibration absorber based on inerter in Fig. 16(b). Main influence of inerter in vibration system in Fig. 16(b) is natural frequency reduction. The angular frequencies at peaks are natural frequencies and can be obtained by Eq. (56). And angular frequencies at valleys are antiresonance frequencies. The value of antiresonance frequencies can be calculated by “$\sqrt{k_{2}/\left(m_{2}+b\right)}$”.

**Fig. 18:**
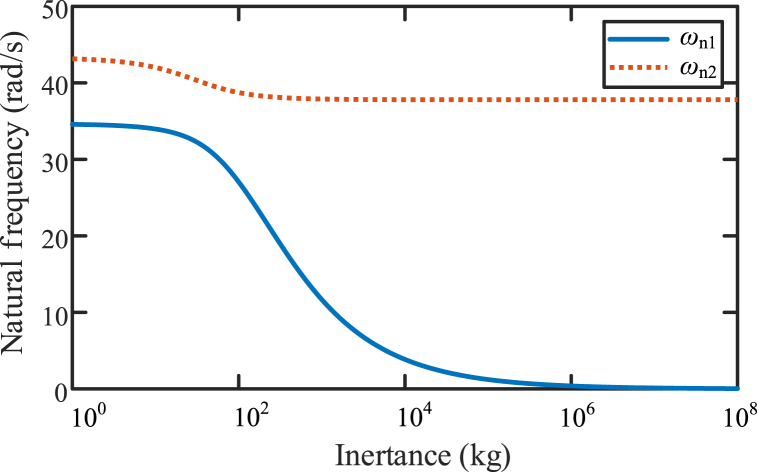
The natural frequencies influenced by inertance of inerter.

Natural frequencies of vibration system with a dynamic vibration absorber based on inerter in Fig. 16(b) influenced by inertance of inerter, is shown in Fig. 18. Blue line is *ω*_n1_ influenced by inertance and red dashed line is *ω*_n2_ influenced by inertance. The two natural frequencies decrease as the inertance increases. As the inertance increases, *ω*_n1_ is convergent to zero, and *ω*_n2_ is convergent to the value of “$\sqrt{k_{1}/\left(m_{1}+m_{2}\right)}$”.

To control vibration using inerter-based vibration absorbers, optimal design is an important aspect. About this aspect, some researches, such as [232,233], are demonstrated effective. Among these, the inerter is equivalent to mass considering its different installation locations. Because of diverse structures of dynamic vibration absorbers based on inerters and categories of applications, the influences of inerters are different and need specific analysis. And the damped case is more complex, there are questions of parameters optimization and structures design of the dynamic vibration absorbers based on inerters. Parameters optimization of dynamic vibration absorbers based on inerters are different also and ought to be based on specific vibration system and analysis.

### 3.3 Vibration isolators based on inerters

Vibration isolators based on inerters [9,41,60,136,147,225,[234], [235], [236], [237], [238], [239], [240], [241], [242], [243], [244], [245], [246], [247], [248], [249], [250], [251], [252], [253], [254], [255], [256], [257], [258]] is another kind of common inerter-based vibration control systems. The vibration isolators based on inerters are mainly applied in vehicle suspension system, the structure vibration control system of buildings, vibration control system of rotating machines, linear and nonlinear energy sink, etc. There are two fundamental kinds of vibration isolations: active vibration isolation as well as passive vibration isolation. And in the question of passive vibration isolation, vibration source is displacement excitation of foundation, and the objective is to reduce vibration transmitted to main system. In the question of active vibration isolation, vibration source is force excitation of main system, and the objective is to reduce vibrational force transmitted to foundation.

**Fig. 19:**
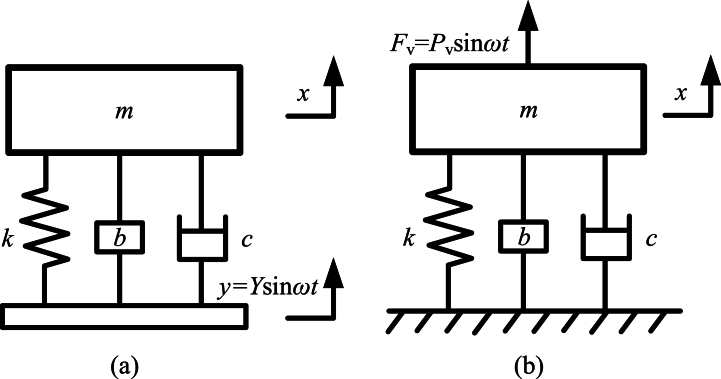
Two kinds of typical vibration isolators based on inerters [234].

The two kinds of typical vibration isolators based on inerters [234] are shown as Fig. 19. And Fig. 19(a) shows typical passive vibration isolation based on inerter. Fig. 19(b) shows the typical active vibration isolation based on inerter. In Fig. 19(a), the simple harmonic exciting displacement of foundation is denoted as *y*. And, exciting displacement *y* can be expressed as “*Y*sin*ωt*”. *Y* is amplitude of exciting displacement *y* and *ω* is angular frequency of exciting displacement *y*. In Fig. 19(b), simple harmonic exciting force applied to the mass block is denoted as *F*_v_. And, the exciting force *F*_v_ can be expressed as “*P*_v_sin*ωt*”. *P*_v_ is amplitude of exciting force *F*_v_. *ω* is angular frequency of exciting force *F*_v_.

The dynamic relationships in the typical passive vibration isolation based on inerter in Fig. 19(a) are expressed as Eq. (57).

(57) $$m\overset{‥}{x}+b\left(\overset{‥}{x}-\overset{‥}{y}\right)+k\left(x-y\right)+c\left(\dot{x}-\dot{y}\right)=0$$

Denote relative displacement *x*-*y* as *z*, and substitute “*y* = *Y*sin*ωt*” into Eq. (57), Eq. (58) is obtained.

(58) $$\left(m+b\right)\overset{‥}{z}+kz+c\dot{z}=m\omega^{2}Y\;\sin\;\omega t$$

Considering the steady-state solution of Eq. (58), the simple harmonic exciting displacement *y* can be expressed as “*Y*e^i*ωt*^”. Complex amplitude of relative displacement *z* is $\bar{Z}$. Complex amplitude of *z* is expressed as “$z=\bar{Z}e^{i\omega t}$”. Based on Eq. (58), complex amplitude of *z* is obtained and shown as Eq. (59).

(59) $$\bar{Z}=\frac{m\omega^{2}Y}{k-\left(m+b\right)\omega^{2}+ic\omega }$$

The complex amplitude of *x* is denoted as $\bar{X}$ and is expressed as “$x=\bar{X}e^{i\omega t}$”. Based on Eq. (59), complex amplitude of *x* is obtained and shown as Eq. (60).

(60) $$x=\bar{X}e^{i\omega t}=z+y=\frac{\left(k-b\omega^{2}+ic\omega \right)Ye^{i\omega t}}{k-\left(m+b\right)\omega^{2}+ic\omega },\bar{X}=\frac{\left(k-b\omega^{2}+ic\omega \right)Y}{k-\left(m+b\right)\omega^{2}+ic\omega }$$

The transmissibility of the displacement *x* and the exciting displacement *y* is *T* and is shown as Eq. (61).

(61) $$T=\left|\frac{x}{y}\right|=\frac{\left|\bar{X}\right|}{Y}=\sqrt{\frac{{\left(k-b\omega^{2}\right)}^{2}+c^{2}\omega^{2}}{{\left[k-\left(m+b\right)\omega^{2}\right]}^{2}+c^{2}\omega^{2}}}$$

The dynamic relationship in the typical active vibration isolation based on inerter in Fig. 19(b) is shown as Eq. (62).

(62) $$\left(m+b\right)\overset{‥}{x}+kx+c\dot{x}=P_{v}\;\sin\;\omega t$$

Considering the steady-state solution of Eq. (62), the simple harmonic exciting force *F*_v_ can be expressed as “*P*_v_e^i*ωt*^”. Complex amplitude of displacement *x* is $\bar{X}$, as mentioned above. Complex amplitude of *x* is expressed as “$x=\bar{X}e^{i\omega t}$”. Based on Eq. (62), complex amplitude of *x* is obtained and shown as Eq. (63).

(63) $$\bar{X}=\frac{P_{v}}{k-\left(m+b\right)\omega^{2}+ic\omega }$$

The transmissibility of vibrational force transmitted to foundation and exciting force *F*_v_ is denoted as *T* and is shown as Eq. (64).

(64) $$T=\left|\frac{b\overset{‥}{x}+kx+c\dot{x}}{F_{v}}\right|=\left|\frac{k-b\omega^{2}+ic\omega }{k-\left(m+b\right)\omega^{2}+ic\omega }\right|=\sqrt{\frac{{\left(k-b\omega^{2}\right)}^{2}+c^{2}\omega^{2}}{{\left[k-\left(m+b\right)\omega^{2}\right]}^{2}+c^{2}\omega^{2}}}$$

It can be seen that the transmissibility *T* in Eq. (61) has the same expression with it in Eq. (64).

Natural frequency of primary vibration system without inerter in Fig. 19 is *ω*_n0_, and is shown as Eq. (65). The frequency ratio can be expressed as “*ω*/*ω*_n0_”.

(65) $$\omega_{n0}=\sqrt{\frac{k}{m}}$$

**Fig. 20:**
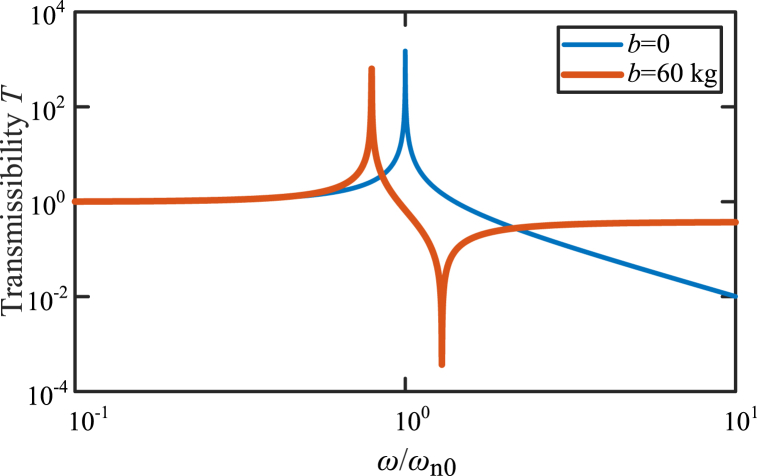
Transmissibility of the typical vibration isolations based on inerter.

Considering the undamped case, and the parameters are that: *m* = 100 kg, *b* = 60 kg, *k* = 4 × 10^3^ N/m, *c* = 0. The curves of the transmissibility *T* versus the frequency ratio *ω*/*ω*_n0_ in the vibration systems shown in Fig. 19, are shown in Fig. 20. Thin blue line is transmissibility *T* of a vibration system without inerter in Fig. 19. Thick red line is transmissibility *T* of a vibration system with two kinds of vibration isolators based on inerters in Fig. 19. The main influence of the inerter in vibration system in Fig. 19 is natural frequency reduction, antiresonance and high-frequency performance degradation. The angular frequencies at the peaks are natural frequencies and can be calculated by “$\sqrt{k/\left(m+b\right)}$”. And the angular frequency at the valley is the antiresonance frequency. The value of the antiresonance frequency can be calculated by “$\sqrt{k/b}$”.

Similar with the dynamic vibration absorbers based on inerters, because of the diverse structures of the vibration isolators based on inerters and categories of applications, the influences of inerters are different and need specific analysis. And the damped case is more complex, there are questions of parameters optimization and structures design of the vibration isolators based on inerters. Parameters optimization of vibration isolators based on inerters are different also and ought to be based on specific vibration system and analysis.

## 4 Conclusions

Inerter is a two-terminal dynamic element proposed in 2002, based on analogy theory between mechanical system and electric system. Inerter has made significant progress in vibration control systems, and researches of the inerter increase by years. Categories of inerters were applied in different systems, such as the vehicle suspension system, the structure vibration control system of buildings, vibration control system of bridges, the landing gear buffer system, vibration energy harvesting device and energy sink, etc. Inerter has provided different routes to the vibration control problems of these systems, compared with the traditional solutions. And inerters have been proved to be effective in many applications. This paper is a review about the physical realizations of inerter and inerter-based vibration control. First, develop of inerter and typical physical realizations of inerter are introduced. The normative derivation processes based on Lagrange equation method of the dynamic relationships in the different inerters are summarized. And then, three categories of common inerter-based vibration control systems are explained. The trend of the physical realizations of inerter, and the possible researches on inerter-based vibration control are summarized as follows.

1. Inerter is developed based on analogy theory between mechanical system and electric system. In the past 20 years, various physical realizations of inerters have appeared. Physical realizations of inerter are different physical devices used for specific mechanical systems. The dynamic characteristics and dynamic models of different physical realizations of inerter vary greatly. The characteristics of rack-and-pinion inerter, as well as ball-screw inerter, hydraulic inerter, fluid inerter, living-hinge inerter, electromechanical inerter, crank inerter and yoke-type nonlinear inerter are explained. Physical realizations of inerter will be more diverse, with different performances and novel structures. And the electromechanical and nonlinear inerters may will be research emphases.
2. Inerter has provided different routes to vibration control problems of mechanical systems. Inerters have been proved to be effective in many applications. Three categories of common inerter-based vibration control systems: the energy dissipators based on inerters, the dynamic vibration absorbers based on inerters and the vibration isolators based on inerters, are explained. There will be more categories of inerter-based vibration control systems. Parameters optimization methods and structure design of the inerter-based vibration control will continue to be research emphases. The systems with inerters are becoming more and more complex, from linear to nonlinear.
3. Inerter is a basic two-terminal element. Dynamic characteristic of ideal inerter is pure inertia. It is quite important that inerter is not a device with single function or single effect. The influence of the applications of inerters depend on the designers' demand and the characteristics of the mechanical system. Inerters will continue to provide solutions to difficult problems in civil engineering, mechanical engineering, etc.

## Data availability statement

Data associated with the study was not been deposited into a publicly available repository. No data was used for the research described in the article.

## CRediT authorship contribution statement

**Yuehao Li:** Writing – original draft, Methodology, Conceptualization, Software. **Niaoqing Hu:** Supervision, Investigation, Visualization. **Yi Yang:** Writing – review & editing. **Zhe Cheng:** Supervision. **Zhengyang Yin:** Software, Validation. **Zuanbo Zhou:** Investigation, Visualization. **Jiangtao Hu:** Investigation, Validation.

## Declaration of competing interest

The author(s) declared no potential conflicts of interest with respect to the research, authorship, and/or publication of this article.
